# Results on SARS-CoV-2 mRNA Vaccine Booster from an Open-Label Multicenter Study in Ofatumumab-Treated Participants with Relapsing Multiple Sclerosis

**DOI:** 10.3390/vaccines11050978

**Published:** 2023-05-13

**Authors:** Tjalf Ziemssen, Eugen Schlegel, Marie Groth, Benjamin Ettle, Tobias Bopp

**Affiliations:** 1 Department of Neurology, Center of Clinical Neuroscience, Carl Gustav Carus University Clinic, University Hospital of Dresden, Technische Universität Dresden, 01062 Dresden, Germany; 2 Zentrum für Neurologische Studien, 57076 Siegen, Germany; schlegel@zns-siegen.de; 3 Novartis Pharma GmbH, 90429 Nuremberg, Germany; marie.groth@novartis.com (M.G.); benjamin.ettle@novartis.com (B.E.); 4 Institute for Immunology, University Medical Center, Johannes Gutenberg University, 55131 Mainz, Germany; boppt@uni-mainz.de

**Keywords:** COVID-19 vaccination, relapsing multiple sclerosis, ofatumumab, neutralizing antibodies, T-cell responses

## Abstract

Background: Few data exist on how ofatumumab treatment impacts SARS-CoV-2 booster vaccination response. Methods: KYRIOS is an ongoing prospective open-label multicenter study on the response to initial and booster SARS-CoV-2 mRNA vaccination before or during ofatumumab treatment in relapsing MS patients. The results on the initial vaccination cohort have been published previously. Here, we describe 23 patients who received their initial vaccination outside of the study but booster vaccination during the study. Additionally, we report the booster results of two patients in the initial vaccination cohort. The primary endpoint was SARS-CoV-2-specific T-cell response at month 1. Furthermore, serum total and neutralizing antibodies were measured. Results: The primary endpoint was reached by 87.5% of patients with booster before (booster cohort 1, N = 8) and 46.7% of patients with booster during ofatumumab treatment (booster cohort 2, N = 15). Seroconversion rates for neutralizing antibodies increased from 87.5% at baseline to 100.0% at month 1 in booster cohort 1 and from 71.4% to 93.3% in booster cohort 2. Of note, 3 of 4 initially seronegative patients in booster cohort 2 and one seronegative patient in the initial vaccination cohort seroconverted after the booster during ofatumumab treatment. Conclusions: Booster vaccinations increase neutralizing antibody titers in ofatumumab-treated patients. A booster is recommended in ofatumumab-treated patients.

## 1. Introduction

Health authorities highly recommend vaccination against the severe acute respiratory syndrome coronavirus 2 (SARS-CoV-2) to prevent severe courses of coronavirus disease 2019 (COVID-19). The Robert Koch Institute currently recommends two initial vaccine applications followed by a third vaccination four weeks after the second dose for immunocompromised persons [1]. A fourth vaccination is recommended for vulnerable people [2].

mRNA vaccines against SARS-CoV-2 have been shown to be safe in vulnerable, immunocompromised patient populations, e.g., oncologic patients. Accordingly, a large cohort study including 74,878 patients with active cancer or a history of cancer has found a low rate of vaccination-related adverse events [3]. Regarding the effectiveness of SARS-CoV-2 vaccinations, analyses of immune responses in vulnerable patients suggest relevant B- and T-cell reactivity [4]. However, seroconversion rates after the first vaccination have been found to be far lower than in healthy controls but increased after the second vaccination [5]. In line with these findings, studies on SARS-CoV-2 vaccination in immunocompromised transplant recipients suggest an impaired response towards SARS-CoV-2 initial vaccination with increasing serologic responses after three or more doses of vaccine [6]. Patients with multiple sclerosis (MS) also belong to the group of vulnerable people as treating their disease requires immunomodulating disease-modifying therapies (DMTs).

Ofatumumab, a human anti-CD20 monoclonal antibody for monthly subcutaneous application, is approved in Europe for active relapsing MS (RMS) in adults. Its mode of action involves the selective depletion of CD20+ B-cells but spares CD20-negative long-lived plasma cells. While the first are major contributors to the adaptive immune system, the latter are especially important for immune memory [7].

For B-cell depleting therapies other than ofatumumab, it is recommended to wait at least three to six months after the last injection before vaccines are applied. This is not very compatible with the recommended regimen of SARS-CoV-2 mRNA vaccination. The interruption of treatment or delayed initiation of treatment should be avoided because of the risk of disease progression [8,9]; however, it is important to assess whether vaccination under continuous ofatumumab therapy elicits an immune response.

According to a retrospective chart review, seroconversion for SARS-CoV-2 neutralizing antibodies is impaired in MS patients receiving anti-CD20 antibodies, with lower seroconversion rates with rituximab and ocrelizumab compared to ofatumumab [10]. In line with these retrospective results, it has recently been shown in a prospective setting that ofatumumab-treated patients respond to initial vaccination against SARS-CoV-2 and while neutralizing antibody titers were reduced under ofatumumab, T-cell response was not affected [11,12]. SARS-CoV-2 infections after two doses of vaccine in patients receiving anti-CD20 antibodies were reported to be mild or asymptomatic and almost all patients seroconverted after infection [13]. Similarly, it has been suggested that booster vaccinations are of particular relevance for increasing antibody titers [14]. Regarding T-cell responses, conflicting findings either suggest no significant effect on T-cell levels [14] or the induction of memory T-cell levels after booster vaccinations [15]. Moreover, there are still few data on how patients with MS receiving treatment with ofatumumab respond to booster vaccination. Some data collected in patients receiving injectable anti-CD20 antibodies suggest a significant increase in antibody titers after a third SARS-CoV-2 vaccination [16]. Similar results were found in an observational study including patients with ofatumumab treatment. In that study, the third vaccination increased IgG titers by a factor of 1.4 to 1.6 compared to titers after the second vaccination. This study also suggests that after three vaccinations, during ofatumumab treatment, B-cell and T-cell responses were still lower than among healthy controls and compared to patients who received two or three doses of vaccine before ofatumumab was started [17].

The objective of the KYRIOS study was to examine the immune response after the completion of initial vaccination against SARS-CoV-2 with mRNA vaccines as well as the immune response to a booster SARS-CoV-2 mRNA vaccine. Therefore, the presence of SARS-CoV-2-specific T-cells and neutralizing as well as total antibodies was analyzed in the KYRIOS study. We here present the results in the subpopulation of patients who received a booster vaccination. The results on the initial vaccination population have been reported previously [11].

## 2. Materials and Methods

Details on study design, participants, treatments, outcomes and assessments as well as statistical analyses have been published previously [11]. Briefly, KYRIOS is a prospective, open-label, multicenter study (EudraCT 2021-000307-20; NCT04869358) designed to investigate the immune response towards SARS-CoV-2 mRNA vaccines in RMS patients in whom ofatumumab has already been initiated or who are planned to be initiated on ofatumumab upon the physician’s discretion.

**Figure 1:**
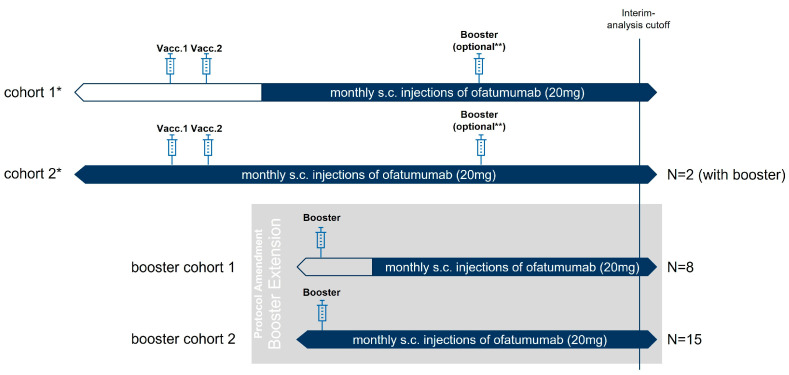
Description of different cohorts in KYRIOS. Cohort 1 and cohort 2 form the initial vaccination cohorts, i.e., patients who received the first and second dose of the SARS-CoV-2 vaccine during the KYRIOS study, either before or during ofatumumab treatment. Two patients of cohort 2 have already received a booster vaccination within the KYRIOS study. Booster cohort 1 and booster cohort 2 consist of patients who received only their booster vaccination during the KYRIOS study (but not the initial vaccination), either before or during ofatumumab treatment. Month 1 results after booster vaccination are reported for booster cohort 1, booster cohort 2, and for the two patients of the initial cohort 2. * Week 1 and month 1 results after the initial vaccination in cohort 1 (N = 6) and cohort 2 (N = 5) have been reported previously [11]. ** Booster vaccinations were optional in cohort 1 and cohort 2 and can be performed any time after the second dose of SARS-CoV-2 vaccine.

The original study protocol comprised cohort 1 and cohort 2, which included patients who received their initial vaccination (i.e., the first and second doses of SARS-CoV-2 mRNA vaccines) during the study either before ofatumumab was started or during stable ofatumumab therapy (Figure 1). Stable ofatumumab treatment was defined as treatment that had been started at least four weeks ago. The depletion of B-cells was verified before vaccination. The week 1 and month 1 results of cohort 1 and cohort 2 have been published previously [11]. Booster vaccinations in these cohorts were optional. We here report the booster vaccination results of 2 patients in cohort 2 (Figure 1).

A protocol amendment introduced booster cohorts 1 and 2 to the study. These cohorts included patients who had already completed the initial vaccination cycle outside the study and received only their booster vaccine during the study (Figure 1). Booster cohort 2 patients may have received their initial vaccination before or during ofatumumab treatment. We here report results of booster cohort 1 and 2.

An additional cohort (cohort 3) with patients from the AMA-VACC study was included as the historical control group. These patients were booster vaccinated while receiving treatment with dimethyl fumarate (DMF), glatiramer acetate (GA), beta-interferons (IFN), or teriflunomide (TF) or while not being treated with a DMT [18].

The primary endpoint was the proportion of patients with SARS-CoV-2-specific T-cell response one month after completion of the booster vaccination. T-cell response was defined as the presence of SARS-CoV-2-reactive T-cells secreting either IFN-γ or IL-2 or both (any T-cell activity above the basal level). Secondary endpoints included the following: the extent of T-cell response defined as T-cell reactivity normalized for basal T-cell activity measured by IFN-γ secretion (IFN-γ stimulation indices); the proportion of patients with neutralizing antibodies against SARS-CoV-2; titers of serum total and neutralizing antibodies against SARS-CoV-2; the incidence of COVID-19 after complete vaccination; and comparison of immune responses in KYRIOS cohorts with the responses in cohort 3 derived from AMA-VACC.

Assessment time points for the booster cohorts in the KYRIOS study were month 1, month 6, month 12 and month 18. We present a pre-planned interim analysis of data obtained one month after the booster vaccination (data cut-off: 12 July 2022). The study is ongoing and follow-up data collected at further time points will be presented upon study completion. All endpoints were analyzed descriptively without formal statistical testing.

The study is consistent with the Declaration of Helsinki and conducted according to the Good Clinical Practice guidelines by the International Conference on Harmonisation (ICH-GCP). Ethics committee approval was obtained, and all patients or their legal representatives provided written informed consent before any study-related procedures were started.

Of note, recruitment into this study started in May 2021, which was before the Robert Koch-Institute issued its recommendation for a third vaccination as soon as four weeks after the second vaccination in severely immunocompromised patients in September 2021 [1]. Only in December 2021 was a joint statement published by the Deutsche Multiple Sklerose Gesellschaft (DMSG), the Kompetenznetz Multiple Sklerose (KKNMS) and the Berufsverband Deutscher Neurologen (BDN) which specified that this recommendation should be applied to MS patients receiving anti-CD20 antibodies or S1P-inhibitors [19].

## 3. Results

The KYRIOS study included 23 patients who received their initial vaccination (first and second dose) outside the study and their booster vaccination during the study. All these patients were initially vaccinated before ofatumumab treatment was started; eight patients also had their booster vaccine before starting ofatumumab (booster cohort 1) while fifteen patients received their booster while being continuously treated with ofatumumab (booster cohort 2). Furthermore, data from two patients in the initial cohort 2 are available, who received their initial and their booster vaccination during the study, both while being treated with ofatumumab.

Table 1 shows the patient characteristics of the booster population at screening. Briefly, patients in booster cohort 1 were on average 47.1 years of age and 45.5 years of age in booster cohort 2. The disease was diagnosed on average 11.1 and 7.2 years ago, respectively. In total, 37.5% and 33.3% of patients were previously untreated in booster cohort 1 and booster cohort 2, respectively. Mostly, mRNA vaccines by BioNTech/Pfizer were administered as the booster, with an average of 26 weeks after the second dose both in booster cohort 1 and booster cohort 2. The mean time between booster vaccination and the start of ofatumumab in booster cohort 1 was 0.87 months. In booster cohort 2, ofatumumab was started on average 1.87 months before booster vaccination (Table 2). Cohort 3 included 20 patients from the AMA-VACC study treated with DMF, GA, IFN or TF, of whom 18 patients received a booster vaccination. The patient characteristics of the AMA-VACC population are presented in Table 1, as published previously [18]. In total, 62.5% of booster cohort 1 and 66.7% of booster cohort 2 had received DMTs prior to ofatumumab. In most cases, this treatment was continued during initial vaccination. Overall, 37.5% patients in booster cohort 1 and 46.7% patients in booster cohort 2 received DMTs during their initial vaccination.

**Figure 2:**
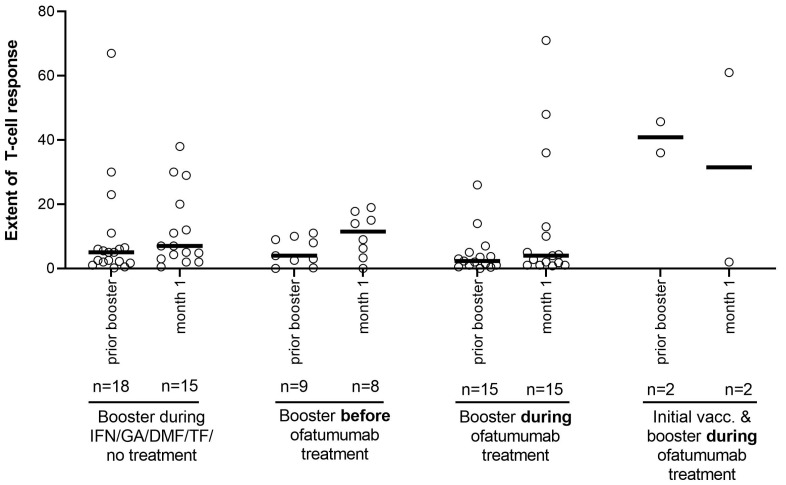
ELISpot-based quantification of T-cell reactivity after booster vaccination prior to or during ofatumumab treatment by calculation of IFN-γ stimulation indices towards SARS-CoV-2. Each dot represents one patient; medians are indicated by horizontal lines. All patients received their initial vaccination cycle before starting ofatumumab treatment (except for 2 patients with initial and booster during ofatumumab treatment). DMF: dimethyl fumarate; GA: glatiramer acetate, IFN: interferon-beta; n: number of patients with assessments; TF: teriflunomide.

We observed a T-cell response according to the primary endpoint definition (presence of SARS-CoV-2 reactive T-cells secreting either IFN-γ or IL-2 or both) in 87.5% of patients in booster cohort 1 and in 46.7% of patients in booster cohort 2. The extent of T-cell response (T-cell reactivity measured by IFN-γ secretion normalized for basal T-cell activity, i.e., IFN-γ stimulation indices) was not markedly different between the two booster cohorts (Figure 2).

**Figure 3:**
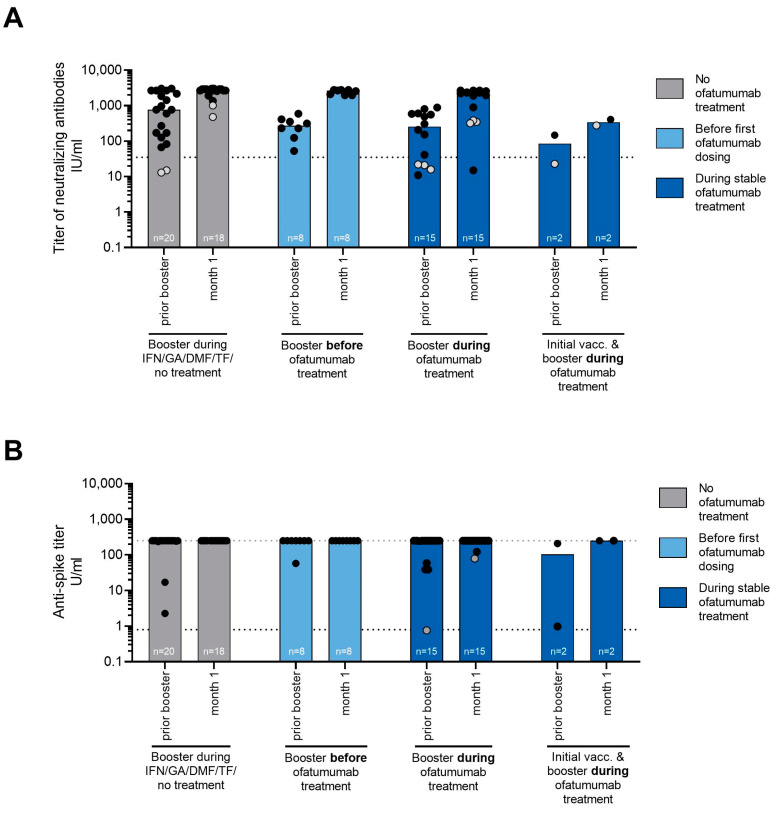
(**A**) Quantification of SARS-CoV-2-specific neutralizing antibody titer in U/mL after booster vaccination prior to or during ofatumumab treatment. (**B**) SARS-CoV-2-specific serum total antibody titer in U/mL after booster vaccination prior to or during ofatumumab treatment. All patients with available data were included in the analysis and individual values are represented by dots. Grey dots = patients who seroconverted after booster. Bars show median values, black dotted lines indicate assay-specific cut-off for seropositivity and grey dotted line indicates the maximal value of quantification range. DMF: dimethyl fumarate; GA: glatiramer acetate, IFN: interferon-beta; n: number of patients with assessments; TF: teriflunomide.

Neutralizing antibody titers increased in booster cohort 1 and 2. The seroconversion rate in booster cohort 1 was 87.5% prior to and 100.0% after the booster in booster cohort 1, and increased from 71.4% prior to the booster to 93.3% at month 1 after the booster in booster cohort 2. Patients in booster cohort 2 with prevalent antibodies before the booster reached similar titers as booster cohort 1 and 3. Of note, of the four patients of booster cohort 2 who were seronegative after the initial vaccination, three patients reached seroconversion after booster vaccination. Of the two patients who had both their initial and booster vaccination during the study while receiving ofatumumab treatment, one patient was seropositive for neutralizing antibodies already prior to the booster and showed an increase in antibody titers at month 1. The second patient was seronegative before and seroconverted after the booster vaccination. In cohort 3, all but two patients were seropositive before the booster. Both patients were seropositive after the booster vaccination (Figure 3A).

Before the booster vaccination, all but three patients (one in booster cohort 1; two in cohort 3) had SARS-CoV-2 serum total antibody titers of 250 U/mL (assay-specific maximum of quantification range). After the booster vaccination, all patients in both booster cohorts had reached titers of 250 U/mL. In booster cohort 2, all but four patients had SARS-CoV-2 serum total antibody titers of 250 U/mL before the booster. In all these patients, total anti-spike antibody titers increased after booster vaccination. Of note, one patient in booster cohort 2 was seronegative for total antibodies before and seroconverted after the booster vaccination. Furthermore, of the two patients with initial and booster vaccination during the study while receiving ofatumumab, both reached serum total antibody titers of 250 U/mL after their booster (Figure 3B).

The median time of observation in the study until the data cut-off was 30.9 weeks. During this period, adverse events (AEs) were reported in 19 patients (82.6%), with 7 cases being related to the DMT and 2 cases being related to the vaccine, i.e., fatigue and tinnitus, which have both been reported as mild (Table 3). Only one relapse in one patient occurred (booster cohort 1). No serious AEs and no deaths were observed.

During the observational period, six cases of clinical COVID-19 were reported, one in booster cohort 1 and five in booster cohort 2. The infections occurred two months after the second vaccination in booster cohort 1, and two months (one patient), three months (one patient), as well as four months (three patients) after the second vaccination in booster cohort 2. All infections were mild or moderate according to CTCAE grading with full recovery in all cases. In booster cohort 1, the infection lasted 7 days, while the duration of infection in cohort 2 ranged from 8 to 14 days.

## 4. Discussion

We here report the results on the subpopulation of patients who received a booster vaccination in the KYRIOS study. The results on the initial vaccination population have been published previously [11]. The present results therefore add to the previous results and provide essential insights on the immune response to booster vaccinations before and during ofatumumab therapy.

Accordingly, T-cell reactivity towards SARS-CoV-2 vaccines showed a slight increase in all booster cohorts at month 1 after the booster vaccination, irrespective of whether patients were vaccinated prior to ofatumumab or during continuous ofatumumab or whether they received other DMTs when vaccinated.

As far as T-cell response is concerned, it has been previously shown by several analyses that anti-CD20 treatment including ofatumumab does not alter T-cell reactivity towards SARS-CoV-2 [11,12,20]. The fact that T-cell reactivity only showed a minor increase in our analysis of booster vaccinations might be an issue of timing of assessments. T-cell reactivity becomes difficult to detect at month 1, as T-cells specific for SARS-CoV-2 then no longer circulate in the blood and tissue-resident memory T-cells develop [21]. It can be assumed that in patients receiving a booster vaccination, T-cell reactivity peaks even earlier due to altered immune mechanisms in immunized patients. However, as antibodies were detected, prior T-cell reactivity in the booster cohorts can be assumed, as T-cells are essential for B-cell-mediated antibody formation in response to mRNA vaccines [22]. It has to be noted that booster vaccinations are of special relevance for increasing antibody levels [14].

Booster vaccinations showed a similar humoral response irrespective of whether the booster was applied prior to or during ofatumumab treatment. All patients who had their booster vaccination during ofatumumab treatment showed an increase in neutralizing antibodies comparable to the cohorts vaccinated prior to ofatumumab or under other DMTs. However, while the relative increase was higher in patients with lower levels before the booster, higher pre-booster antibody levels seem to be associated with a higher absolute antibody level after the booster [23]. Antibody levels prior to the booster, in turn, are likely to depend on prior treatment. In the study by Faissner et al., an impaired humoral response was reported [12], but baseline antibody levels have not been assessed. However, three patients were treated with sphingosine-1-phosphate (S1P) inhibitors during their initial SARS-CoV-2 vaccination (i.e., first and second dose of the vaccine) [12]. S1P inhibitors are known to be associated with reduced antibody levels in response to vaccination during continuous treatment [18]. Therefore, prior treatments in the Faissner study might have impacted the antibody development after vaccination against SARS-CoV-2. In the KYRIOS study, seronegative patients or patients with lower titers before the booster vaccination showed almost similar increases, and 75% of seronegative patients before the booster vaccination reached seropositivity at month 1 even when vaccinated during continuous ofatumumab. Of note, two-thirds of the patients in booster cohort 2 had received other DMTs prior to ofatumumab, including S1P-inhibitors. It seems reasonable to assume that these prior treatments might have also affected the immune response to SARS-CoV-2 vaccination, especially when DMTs were switched only shortly before the vaccination.

The fact that booster vaccination further increased neutralizing antibody titers also in those who received their first vaccination during ofatumumab suggests the development of immune memory after their initial vaccination. The application of a booster vaccination then activates humoral immune memory and leads to the proliferation of antigen-specific B-cells. Preclinical data on ofatumumab support these findings. In contrast to ocrelizumab, human equivalent doses of ofatumumab applied in huCD20 transgenic mice were shown to spare marginal zone and follicular B cells in lymphoid organs and in the bone marrow, which are important for immune surveillance, B-cell repletion and preservation of the immune response [24].

Regarding booster vaccinations, the KYRIOS study suggested similar immune responses in patients receiving the booster either prior to or during ofatumumab. It can be assumed that it is not necessary to postpone ofatumumab treatment initiation until after the booster vaccination. Furthermore, booster data indicate that patients who had received their initial vaccination during ofatumumab benefit from an additional booster vaccination. This supports the vaccination recommendations toward a third vaccination as soon as four weeks after the second vaccination in MS patients receiving anti-CD20 antibodies or S1P-inhibitors [1,19]. It can be assumed that as in immunosuppressed transplant recipients, each booster leads to an increase in seroconversion rate [6].

In general, higher antibody titers are correlated with better protection from severe COVID-19. The KYRIOS results suggest clinically effective vaccinations in MS patients receiving ofatumumab, irrespective of whether vaccination is completed before treatment initiation or whether it is applied during ofatumumab treatment, as no severe infections occurred, and infections only lasted 7 to 14 days. Therefore, the severity and duration of COVID-19 in KYRIOS are well in line with ALITHIOS study results on infection in ofatumumab-treated patients [25]. However, as we have previously pointed out [11], COVID-19 cases in ALITHIOS were observed before September 2021, and therefore occurred before the circulation of Omicron. On the contrary, COVID-19 cases in KYRIOS mainly occurred during early 2022. It can be assumed that a relevant number of these cases were Omicron infections. Omicron is known to escape the immune response, but still leaves vaccines effective in preventing severe COVID-19. According to the KYRIOS study, the latter also applies to patients receiving ofatumumab. Given this background and our present data, a booster with the Omicron-adapted vaccine seems reasonable.

SARS-CoV-2 mRNA vaccines were well tolerated in the present study, with only two cases of mild adverse events related to the booster vaccination being reported. No thrombosis or cardiovascular events have been reported in the booster cohorts. Vaccine-induced immune thrombocytopenia and thrombosis (VITT) are a rare but serious issue associated with SARS-CoV-2 mRNA vaccination [26], possibly mediated by anti-PF4 antibodies, which can be found after vaccination but not after COVID-19 infection [27].

When interpreting the results of KYRIOS, some study limitations have to be kept in mind. As KYRIOS only included a small sample, the results should not be overinterpreted as they require further confirmation. As no week 1 assessment was scheduled for patients who received a booster vaccination, information on early T-cell responses after booster vaccination is lacking. Furthermore, patients were included in the study before the relevant authorities issued a recommendation to apply a third dose as early as four weeks after the second. The time between the first and second dose of the vaccination cycle and booster vaccination was therefore longer (median 6 months) in our study than what is currently recommended.

## 5. Conclusions

Overall, the present KYRIOS data add to the previous results on the immune response towards SARS-CoV-2 mRNA vaccination. The previously reported results gave valuable insights into the impact of concurrent ofatumumab treatment during the initial vaccination cycle. Given that booster vaccinations are generally recommended, information on immune responses to booster vaccinations before and during ofatumumab therapy were needed. The novel KYRIOS booster data show that a booster vaccination increases neutralizing antibody titers in patients continuing ofatumumab therapy. A similar antibody titer is achieved as that in patients not undergoing ofatumumab therapy. The present data give the first indications that a booster may possibly lead to seroconversion in patients who were seronegative after initial vaccination and received a booster during stable ofatumumab treatment, regardless of which therapy the patients had received during the initial vaccination, including ofatumumab. Further data are needed to support this assumption. Since higher titers correlate with vaccine effectiveness, a booster is recommended in patients on ofatumumab therapy.

## Figures and Tables

**Table 1:** Demographic and disease characteristics.

Variable *	Booster Cohort 1 Vaccination Prior to Treatment	Booster Cohort 2 Vaccination during Stable Treatment	Cohort 3 Vaccination during IFN/GA/DMF/TF/no DMT
N	8	15	20 ^a^
Age, years	47.1 (14.1)	45.5 (12.4)	48.6 (12.9)
Sex, female, n (%)	5 (62.5)	9 (60.0)	16 (80.0)
Time since diagnosis, years	11.1 (8.7)	7.2 (7.7)	13.99 (10.43)
Number of prior DMTs	2.0 (2.1)	1.3 (1.2)	1.6 (0.7)
Number of DMTs prior to ofatumumab, n (%)			
0	3 (37.5)	5 (33.3)	0 (0.0)
≥1	5 (62.5)	10 (66.7)	18 (100.00)

* If not indicated otherwise, data are presented as mean (SD). ^a^: 18 of 20 patients in the AMA-VACC study had a booster vaccination. DMF: dimethyl fumarate; GA: glatiramer acetate, IFN: interferon-beta; TF: teriflunomide.

**Table 2:** Vaccination characteristics.

Variable	Booster Cohort 1 Vaccination Prior to Treatment	Booster Cohort 2 Vaccination during Stable Treatment	Cohort 3 Vaccination during IFN/GA/DMF/TF/no DMT
N	8	15	20 ^a^
Vaccination, n (%)			
1st (BioNTech/Pfizer|Moderna)	8 (100.0)|0 (0.0)	14 (93.3)|1 (6.7)	19 (95.0)|1 (5.0)
2nd (BioNTech/Pfizer|Moderna)	8 (100.0)|0 (0.0)	14 (93.3)|1 (6.7)	19 (95.0)|1 (5.0)
First booster vaccination (BioNTech/Pfizer|Moderna)	7 (87.5)|1 (7.1)	13 (65.0)|2 (10.0)	11 (61.1)|7 (38.9)
Vaccination time interval, mean (SD)			
1st to 2nd vaccination, weeks/days	5.6 (0.7) weeks	5.6 (1.4) weeks	36.8 (9.0) days
2nd vaccination to booster vaccination, weeks/months	26.0 (2.3) weeks	26.2 (5.9) weeks	5.82 (0.4) months
Time interval between start of ofatumumab and vaccination, mean (SD)			
Booster vaccination to start of ofatumumab, months	0.87 (0.18)	-	-
Start of ofatumumab to booster vaccination, months	-	1.87 (0.85)	-

^a^: 18 of 20 patients in the AMA-VACC study had a booster vaccination. DMF: dimethyl fumarate; GA: glatiramer acetate, IFN: interferon-beta; TF: teriflunomide.

**Table 3:** Overview of adverse events.

Adverse Events, n (%)	Booster Cohort 1 Booster Vaccination Prior to Treatment (N = 8)	Booster Cohort 2 Booster Vaccination during Stable Treatment (N = 15)
Adverse events (AEs)	6 (75.0)	13 (86.7)
General disorders and administration site conditions	1 (12.5)	3 (20.0)
Nervous system disorders	3 (37.5)	4 (26.7)
Musculoskeletal and connective tissue disorders	2 (25.0)	2 (13.3)
Blood and lymphatic system disorders	0 (0.0)	0 (0.0)
Infections and infestations	3 (37.5)	9 (60.0)
Ear and labyrinth disorders	0 (0.0)	1 (6.7)
Gastrointestinal disorders	1 (12.5)	0 (0.0)
Injury, poisoning and procedural complications	2 (25.0)	1 (6.7)
Metabolism and nutrition disorders	1 (12.5)	0 (0.0)
Psychiatric disorders	0 (0.0)	1 (6.7)
Reproductive system and breast disorders	2 (25.0)	0 (0.0)
Respiratory, thoracic and mediastinal disorders	1 (12.5)	0 (0.0)
Skin and subcutaneous tissue disorders	1 (12.5)	1 (6.7)
Vascular disorders	0 (0.0)	2 (13.3)
Not coded	2 (25.0)	2 (13.3)
AEs related to DMTs	4 (50.0)	3 (20.0)
AEs related to SARS-CoV-2 vaccine	0 (0.0)	2 (13.3)
AEs leading to permanent discontinuation of study medication	0 (0.0)	0 (0.0)
AEs leading to temporary interruption of study medication	0 (0.0)	1 (6.7)
Serious adverse events	0 (0.0)	0 (0.0)

In case of multiple AEs, a patient is counted only once in the respective category.

## Data Availability

Data will be provided upon reasonable request.
